# New Instrument for Making Extension in Cases of Dislocation of Small Joints

**Published:** 1847-04

**Authors:** F. H. Hamilton


ART. V.—*New instrument for making extension in cases of dislocation of small Joints.* By F. H. HAMILTON, M. D.

DR. FLINT—Dear Sir:—I have in my possession an instrument for making extension in cases of dislocation of small joints, which is, I believe, superior to any thing which has ever before been invented or used.

I scarcely know how to describe it so as to be intelligible, yet it is very simple. It is made by the Indians in this vicinity, and may always be had during the summer, at the Indian Toy Shops at Niagara Falls. The Indians call it a "puzzle." and know no other use for it than to fasten it upon the thumb or finger of some victim, and then pull him about until he begs to be released.

The Indians in Maine, I am told, make the same thing also, and for the same purpose.

The "puzzle" is an elongated cone of about 6 or 8 inches in length, made of ash splittings and braided ; the open end of the cone being about three-fourths of an inch in diameter, and the opposite one terminating in a braided cord.

When applied to the finger it is slipped on lightly, forming a cap to the extremity and half the length of the finger, and on being drawn upon it fastens itself upon the member with a most unflinching grasp, and if of proper size, and made of proper material, it becomes the more securely fastened in proportion as the traction is increased ; yet applying itself equally to all the surfaces it inflicts the least possible amount of pain and injury upon the limb. When you wish to remove it you have only to cease pulling and it drops off spontaneously In reduction of a dislocated thumb where ordinary extension fails, it will be found of invaluable service.

Yours truly,

F. H. HAMILTON.

